# Adult-Onset Phalangeal Microgeodic Syndrome: A Case Report and Literature Review

**DOI:** 10.7759/cureus.80811

**Published:** 2025-03-19

**Authors:** Takeshi Zoshima

**Affiliations:** 1 Internal Medicine, Asanogawa General Hospital, Kanazawa, JPN; 2 Nephrology and Rheumatology, Kanazawa University Hospital, Kanazawa, JPN

**Keywords:** adult-onset, bone lesion, mri images, phalangeal microgeodic syndrome, toe pain

## Abstract

Phalangeal microgeodic syndrome (PMS) is a rare condition thought to result from a transient disturbance of peripheral circulation under cold temperatures. PMS is most prevalent in children, mainly affecting the fingers and rarely the toes. Although some cases of adult-onset PMS have been reported, the clinical features remain unclear. Herein, I report the case of a 24-year-old man who developed PMS with toe involvement. The self-limiting course was followed by magnetic resonance imaging (MRI). Furthermore, I performed a literature review and summarized the characteristics of this case and eight previously reported adult-onset PMS cases (n=9). The median patient age was approximately 43 (range: 18-89) years, and most cases were diagnosed based on frostbite-like symptoms in winter and specific MRI findings, despite normal radiographic findings. While adult-onset cases had similar clinical and imaging features to child-onset cases, toe involvement was frequent (5/9 [56%]), and female patients were dominant (8/9 [89%]). Two patients had immune-mediated diseases, such as systemic lupus erythematosus and psoriasis. These reports suggest that adults and children with PMS have some different clinical characteristics. Thus, clinicians should consider PMS when finger or toe pain occurs in cold environments, even in adults.

## Introduction

Phalangeal microgeodic syndrome (PMS) is a rare condition characterized by pain and swelling of the digits. PMS is most prevalent in children, mainly affecting the fingers and rarely the toes, and typically develops with frostbite-like symptoms in winter [1]. A transient disturbance of peripheral circulation under cold temperatures is thought to trigger PMS [1]. Typical clinical manifestations and imaging findings, such as radiography or magnetic resonance imaging (MRI), are sufficient for diagnosis; thus, invasive examinations are usually unnecessary. PMS is a self-limiting condition with a favorable prognosis, and conservative management is preferred [1]. Symptoms and radiographic abnormalities usually return to normal within 3-12 months without intervention [1,2].

PMS primarily affects children and rarely adults. A previous study that included 161 patients with PMS reported a mean age of approximately four years old; all patients were <15 years old [3]. Nonetheless, some adult cases (i.e., those who developed PMS at age ≥18 years) have been reported [3-9]. However, due to its rarity, the clinical features of adult-onset PMS still require elucidation.

Herein, I report the case of a 24-year-old man who developed PMS with toe involvement. The self-limiting course was followed by an MRI. Moreover, I reviewed previous cases of adult-onset PMS reported in the literature, aiming to identify the differences in clinical characteristics between child- and adult-onset PMS.

## Case presentation

**Figure 1:**
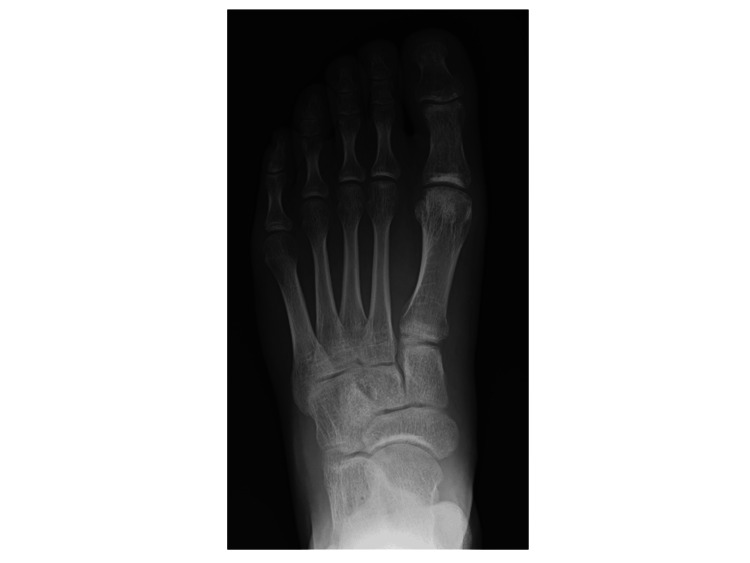
Foot X-ray findings. An anteroposterior radiograph of the left foot shows no abnormalities.

**Figure 2:**
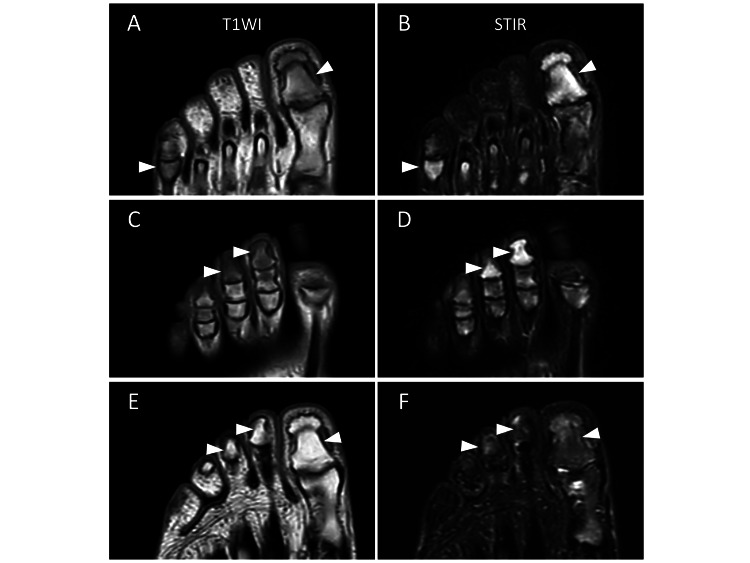
Magnetic resonance imaging (MRI) findings of toes. (A–D) The initial MRI findings. Low-signal intensity on T1-weighted images (T1WI: A and C) and high-signal intensity on short-inversion time-inversion recovery images (STIR: B and D) are observed in the bone marrow of the first, second, and third distal and fifth proximal phalanges of toes (arrowheads). (E–F) The follow-up MRI findings. Significant improvement of the abnormal findings detected in the initial MRI examination is observed (arrowheads). (E) T1WI and (F) STIR.

A 24-year-old man with a history of toe pain for a few months was referred to a rheumatology clinic during the winter. This symptom developed without any triggers, such as trauma, and was exacerbated by weight-bearing and exercise, primarily affecting the first toes. The patient, who noticed redness and purpura in his toes for the first time one month prior, visited an orthopedic clinic and was symptomatically managed as a hallucal sesamoid disorder with no improvement. Treatment of the frostbite-like symptoms at a dermatology clinic abated them, but the toe pain persisted. At the rheumatology clinic, the patient denied having any pain in the fingers or joints other than the toes. He did not take regular medications or have a significant medical history. A physical examination revealed bilateral tenderness, slight swelling, and discoloration of the toes. A blood examination revealed normal C-reactive protein, uric acid, matrix metalloproteinase-3, and angiotensin-converting enzyme levels, and tests for rheumatoid factors and anti-nuclear antibodies, as well as an enzyme-linked immunospot assay, were negative. Plain chest and toe radiographs revealed no abnormalities (Figure 1). However, MRI revealed diffuse low signal intensity on T1-weighted images and high signal intensity on short-inversion time inversion recovery (STIR) images of the bone marrow of the distal and proximal phalanges of some toes (Figures 2A-2D). Therefore, they were diagnosed with PMS. Seven months later (in the summer), the patient’s symptoms and follow-up MRI findings improved significantly without any specific treatment (Figures 2E-2F). 

## Discussion

Here, I report a case of adult-onset PMS with toe involvement; this presentation differs from typical child-onset PMS, which mainly affects the fingers and rarely the toes. Consequently, I reviewed the literature for similar adult-onset PMS cases to determine the clinical characteristics.

I searched the Medline/PubMed database for English-language reports of adults who developed PMS at ≥18 years old until October 2024, identifying eight cases [3-9]. Thus, nine have been reported, including the present case (Table 1). The median patient age was approximately 43 years (range: 18-89), and eight (89%) were female. The present case is the only case involving a male patient. Most cases were diagnosed based on frostbite-like symptoms in the winter and specific MRI findings despite normal radiographic findings. Notably, five (56%) cases had toe involvement and four (44%) had finger involvement. Moreover, two cases were complicated by immune-mediated diseases, such as systemic lupus erythematosus (SLE) and psoriasis. Most cases had a self-limiting course with a favorable prognosis without treatment. However, pentoxifylline therapy was used with success in one case. Therefore, frequent toe involvement, female predominance, and complications of immune-mediated diseases appear to be unique characteristics of adult-onset PMS. 

**Table 1:** Adult-onset phalangeal microgeodic syndrome. *X-ray findings suggestive of phalangeal microgeodic syndrome, including cortical irregularity and multiple foci of radiolucency. **MRI findings suggestive of phalangeal microgeodic syndrome, including diffuse hypointense T1 and hyperintense STIR signal in the phalanges. No: number, NA: not available, MRI: magnetic resonance imaging, STIR: short-inversion time-inversion recovery.

Age, years old	Gender	Lesions		X-ray findings^*^	MRI findings^**^	Complications	Reference
		Fingers	Toes				
38	Female	–	+	+	NA	–	[3]
60	Female	+	–	–	+	NA	[4]
58	Female	+	–	–	+	NA	[4]
50s	Female	+	–	–	+	Systemic lupus erythematosus	[5]
20	Female	–	+	NA	+	NA	[6]
43	Female	–	+	–	+	–	[7]
89	Female	+	–	–	+	Hypertension, chronic heart failure, atrial fibrillation	[8]
18	Female	–	+	NA	+	Psoriasis, major depressive disorder	[9]
24	Male	–	+	–	+	–	The present case

Toe involvement occurs more frequently in adult-onset PMS than in child-onset PMS. A previous study reported that only 18 (8.9%) of 202 child-onset cases involved the toes. The fourth and fifth proximal phalanges of the toes are most frequently affected [10]. Another study reported that none of the 20 child-onset cases had toe involvement [11]. The imaging findings of affected toes are similar to those of the fingers [10,12]. I found that the toes were affected in five (56%) of nine adult-onset PMS cases; thus, frequent involvement of the toes could be unique to adult-onset PMS.

Although no significant sex predilection has been reported for child-onset PMS, female patients were prevalent in adult-onset PMS. A previous study included 123 boys and 79 girls with child-onset PMS [10], and another included 12 boys and eight girls [11]. While both sexes are almost equally affected in children [13], female dominance also appears unique to adult-onset PMS.

Co-existing immune-mediated diseases were also noted in adult-onset PMS cases, specifically SLE and psoriasis. These diseases can cause synovitis, tenosynovitis, dactylitis, or abnormal peripheral circulation, such as Raynaud’s phenomenon, mimicking PMS [5,14]. Owing to the onset of age, immune-mediated disease can be more complicated in adults than in children. Therefore, careful physical and imaging examinations should be used to differentiate these conditions.

MRI may be more useful than radiography for detecting imaging abnormalities in PMS. Originally, PMS was termed because of the specific radiographic findings; microgeodes, small radiolucent spots approximately one millimeter in diameter in the diaphysis of the phalanx [15]. Characteristic MRI findings have been reported, such as low signal intensity on T1-weighted images and high signal intensity on STIR images in the phalanges, suggesting bone marrow edema [1]. These MRI findings can be detected even without X-ray findings [4,16]. While these imaging findings appear to be pathognomonic to PMS [16], some diseases, including multifocal osteomyelitis, tuberculous osteitis, sarcoidosis, osteosarcoma, and Ewing’s sarcoma, should be carefully differentiated using MRI and other clinical findings [2,17]. Therefore, MRI has higher sensitivity [5,6,16] and may detect PMS-related abnormalities earlier than X-ray imaging.

This study has some limitations. First, this was a retrospective case-based review that included only a small number of cases owing to the rarity of adult-onset PMS. Studies including a larger number of such cases are required to confirm these findings. Second, most adult cases were detected based on MRI abnormalities instead of X-ray examination. Thus, studies comparing child-onset PMS diagnosed by abnormal MRI findings may be needed in the future.

## Conclusions

In conclusion, I report a case of an adult-onset PMS with toe involvement, and the self-limiting course was followed by an MRI. Moreover, this literature review suggests that the clinical characteristics differ between adult- and child-onset PMS: frequent toe involvement, female predominance, and complications of immune-mediated diseases. Thus, clinicians should consider PMS when finger or toe pain occurs in cold environments, even in adults.
